# Does Portal Hypertension Increase the Risk of *Helicobacter pylori* Infection and Pre-Malignant Gastric Lesions?

**DOI:** 10.3390/jcm13061768

**Published:** 2024-03-19

**Authors:** Rui Gaspar, Pedro Cardoso, Tiago Ribeiro, Marco Silva, Guilherme Macedo

**Affiliations:** Gastroenterology and Hepatology, Unidade Local de Saúde de São João, Porto 4200, Portugaltiagofcribeiro@outlook.com (T.R.); marcocostasilva87@gmail.com (M.S.); guilhermemacedo59@gmail.com (G.M.)

**Keywords:** chronic liver disease, spleen elastrography, *Helicobacter pylori*, pre-malignant gastric lesions

## Abstract

**Background and Aims:** The presence of portal hypertension in cirrhotic patients is a major prognostic factor associated with the development of severe complications and increased mortality. The gold standard for diagnosing portal hypertension is the hepatic venous pressure gradient. More recently, spleen stiffness has emerged as a new and non-invasive diagnostic tool, and has already been included in the last Baveno VII guidelines. The exact prevalence of *Helicobacter pylori* infection, pre-malignant lesions and their relation to portal hypertension have never been described. The aim of our study was to evaluate the relationship between the presence of portal hypertension assessed via liver and spleen elastography and *Helicobacter pylori* infection and pre-malignant gastric lesions. **Methods:** An observational study was conducted, including consecutive patients admitted from December 2020 to December 2022. All patients underwent upper endoscopy and were also subjected to liver and spleen elastography (using the new probe of 100 Hz) by the same blinded operator in a tertiary center. **Results:** We included 155 cirrhotic patients, with a mean age of 64.1 years (±8.8), and 81.3% were male. The most common etiology was alcoholic liver disease (72.9%). The median value of liver stiffness measurement was 24.4 kPa [3.1–75.0], and the spleen stiffness measurement was 49.1 kPa [12.8–100.0]. Akin to endoscopic findings, 50.3% presented esophageal varices, 5.2% gastric atrophy, 11.6% gastric metaplasia, and 32.9% portal hypertension gastropathy. Regarding histologic findings, we found that 34.8% presented *H. pylori* infection, 35.5% gastric atrophy (OLGA 1—58.2%) and 38.7% gastric metaplasia (OLGIM 1—63.3%). Liver stiffness and spleen stiffness measurements were associated with the presence of portal hypertensive gastropathy (*p* < 0.01), but not with *H. pylori* infection or pre-malignant gastric lesions. **Conclusions**: Although present in almost one third of cirrhotic patients, *H. pylori* infection and pre-malignant gastric lesions are not associated with liver stiffness and spleen stiffness measurements. On the other hand, we found an association between liver stiffness and spleen stiffness measurements and portal hypertensive gastropathy.

## 1. Introduction

Liver cirrhosis is a chronic disease characterized by the progressive accumulation of fibrosis and the development of regenerative nodules, leading to portal hypertension and liver failure [1]. The Global Burden of Diseases, Injuries and Risk Factors Study (GBD) 2017 is the most recent and complete study evaluating the prevalence of liver cirrhosis across the world [2]. It reported a prevalence of more than 120 million cases of cirrhosis worldwide, the vast majority in the compensated phase (>90%) [2]. There are several etiologies for liver cirrhosis, ranging from autoimmune causes (autoimmune hepatitis, primary biliary cholangitis or primary sclerosing cholangitis) and metabolic diseases (non-alcoholic fatty liver disease, alcoholic liver disease, hereditary hemochromatosis, Wilson’s disease and alpha-I antytripsin deficiency) to viral hepatitis (mainly hepatitis B or C) [3].

Cirrhosis is clinically divided into two very different prognostic stages: compensated cirrhosis (or compensated advanced chronic liver disease—cACLD), when there are no episodes of decompensation, with a median survival of more than 12 years; and decompensated cirrhosis, based on the presence of complications related to the development of portal hypertension, showing a marked reduction in median survival (median of 2 years) [4,5].

Portal hypertension is defined as an increase in the hepatic venous pressure gradient (HVPG) above 5 mmHg and it is clinically significant when HVPG ≥ 10 mmHg, as it is independently associated with the development of cirrhosis-related complications such as gastroesophageal bleeding, ascites or hepatic encephalopathy [4,5]. The gold standard for portal hypertension assessment is HVPG measurement through hepatic vein catheterization. However, this procedure is invasive, costly and only performed in specialized centers due to technical difficulties [6,7].

In recent years, there has been intensive research on non-invasive tools for portal hypertension assessment. Liver stiffness measurement (LSM) via liver elastography has emerged as one of the best non-invasive tools for the prediction of the presence of portal hypertension. It has become one of the cornerstones in the Baveno VII guidelines for ruling in or ruling out clinically significant portal hypertension (CSPH) [8,9,10,11]. More recently, spleen stiffness measurement (SSM) via spleen elastography has shown promising results and has increased the diagnostic performance of LSM [12,13]. The development of a novel spleen-dedicated stiffness measurement probe (100 Hz) has further improved the results obtained and holds the potential to become one of the most important non-invasive tools for portal hypertension assessment [4,14].

*Helicobacter pylori (H. pylori)* is a Gram-negative bacillus that mainly resides on the surface of epithelial cells in the stomach [15]. *H. pylori* infection is one of the most common infections worldwide, accounting for more than 50% of cases in developed countries and reaching 90% in developing countries [15]. It is associated with the development of pre-malignant gastric lesions (such as gastric atrophy, metaplasia and dysplasia), peptic ulcers, mucosa-associated lymphoid tissue (MALT) lymphoma and gastric cancer. *H. pylori* is considered one of the most important risk factors for gastric cancer and has been classified as a class I carcinogenic factor by the World Health Organization [16,17]. *H. pylori* has also been found to be involved in extragastrointestinal disorders such as iron-deficient anemia, vitamin B12 deficiency, idiopathic thrombocytopenic purpura, ischemic heart disease or neurodegenerative syndromes [18]. Furthermore, it has been linked to multiple liver diseases such as chronic viral hepatitis and non-alcoholic fatty liver disease (NAFLD). The relation between with *H. pylori* infection and the risk of cirrhosis and portal hypertension is not clear. Some studies reported that *H. pylori* could reduce Il-10 levels (an anti-inflammatory cytokine) and increase the production of IL-1B, IL-8 and adhesion molecules—all factors that contribute to the development of endothelial dysfunction, increasing the risk of portal hypertension [19]. Additional possible mechanisms that could also contribute to the development of portal hypertension include the augmented production of nitric oxide caused by *H. pylori* leading to an increased portal blood flow, and the development of portal collaterals due to the VEGF overexpression induced by *H. pylori* [19,20,21].

A meta-analysis including 13 studies from Asia, Europe and Africa did not find an association between *H. pylori* infection and the presence of esophageal varices [22]. On the other hand, *H. pylori* infection is associated with high levels of ammonia and a higher risk of hepatic encephalopathy [23]. Hepatic encephalopathy is a complex neuropsychiatric syndrome that can be caused by several factors (gastrointestinal bleeding, infection, constipation or hydroelectrolytic disorders) and one of the main causes of admission in cirrhotic patients. The pathophysiology of hepatic encephalopathy is strictly linked to hyperammonemia and it was already established that *H. pylori* increase the levels of ammonia by converting urea into carbon dioxide and ammonia [24]. Moreover, a systemic review showed that *H. pylori* eradication may have an important role in reducing the risk of hepatic encephalopathy in cirrhotic patients [25].

However, a direct relationship between *Helicobacter pylori* infection, pre-malignant lesions and portal hypertension has not yet been described [19,26,27].

The aim of our study was to evaluate the relationship between the presence of portal hypertension, assessed via liver and spleen elastography, and *Helicobacter pylori* infection and pre-malignant gastric lesions.

## 2. Methods

### 2.1. Study Design, Inclusion and Exclusion Criteria

An observational study was conducted on consecutive cirrhotic patients who were willing to participate in our trial and were admitted to the Gastroenterology and Hepatology Department of a tertiary center between December 2020 and December 2022. 

The hospital is located in the city of Porto, the second-largest city of Portugal, and is the major referral center for the population of the north of the country. The Department of Gastroenterology and Hepatology is a tertiary, academic, non-transplant center, and is the largest in the region both in terms of the number of physicians and the number of patients’ referrals.

All patients underwent liver and spleen elastography and esophago-gastroduodenoscopies (EGD) in our department. 

The diagnosis of cirrhosis was made based on either histology or LSM > 12.5 kPa [28].

The inclusion criteria were as follows: (1) an ability to provide informed consent, (2) age > 18 years, and (3) a previous diagnosis of cirrhosis (clinical or histological). 

**Figure 1:**
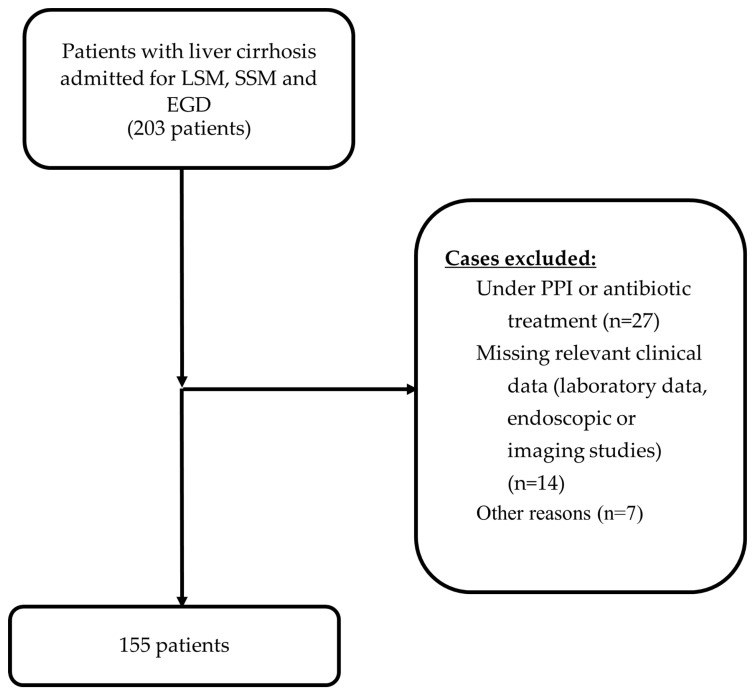
Study flowchart.

Exclusion criteria were as follows: (1) non-cirrhotic portal hypertension, (2) liver transplantation, (3) patients with a transjugular intra-hepatic portosystemic shunt, (4) acute or chronic portal vein thrombosis, (5) liver congestion that is secondary to heart failure, (6) splenectomy or the congenital absence of a spleen, (7) pregnancy, (8) a history of gastric surgery, (9) patients with primary or secondary malignancy, (10) patients who had previously undergone *H. pylori* treatment, (11) treatment with antibiotics, proton pump inhibitors, H2-antagonists, aspirin or anti-inflammatory drugs up to 4 weeks before EGD and LS and SSM, (12) interval between LS and SSM and EGD of more than 6 months, and (13) missing relevant clinical data from the files (Figure 1).

### 2.2. Data Collection

Demographic, laboratory and clinical data were collected from electronic medical records. The etiological diagnosis of cirrhosis included a detailed drug history, the body mass index, serology for HBV and HCV, serum immunoglobulins and a panel of autoantibodies for the diagnosis of autoimmune hepatitis (AIH) or primary biliary cholangitis (PBC) according to established criteria [29,30], as well as the appropriate laboratory data used to diagnose hemochromatosis, Wilson’s disease and α-1 antitrypsin deficiency [31,32,33]. HBV DNA and HCV RNA were further requested to establish the diagnosis of chronic hepatitis B or C, respectively. Alcohol consumption was evaluated by describing the medium quantity and type of drinks consumed. Liver cirrhosis was labelled as cryptogenic when all the available investigations did not lead to or suggest a specific etiology. 

Clinical data collected included the age, sex, race, previous treatment of *Helicobacter pylori* infection, previous gastric surgery, history of alcohol consumption, medication and previous episodes of decompensation. Laboratory data included hemoglobin, white cell and platelet count (PLT), international normalized ratio (INR), albumin, aspartate-aminotransferase (AST), alanine-aminotransferase (ALT), gamma-glutamyl transferase (GGT), alkaline phosphatase (ALP), total and direct bilirubin, urea, creatinine and alpha-fetoprotein (AFP). All patients had imagological evaluation by abdominal ultrasound or MRI for hepatocellular carcinoma screening within 6 months. 

### 2.3. Transient Elastography Examination (LSM and SSM)

The SSM and LSM were performed on the same day with patients in the supine position, with both arms in maximum abduction, with at least 6 h of fasting, using a FibroScan Expert 630 (Echosens^®^, Paris, France) device. SSM and LSM were performed by a single experienced operator who was blinded to the result of the EGD. In cases where EGD and transient elastography were not performed simultaneously, a timespan of less than 6 months was required for inclusion.

LSM was performed with M or XL probes depending on the thickness of the subcutaneous fat, while SSM was performed using a 100 Hz M probe alone. Two separate sets of 10 measurements each were taken for SSM and LSM, and the average value was recorded. Quality criteria applied for SSM measurement were similar to LSM (≥60% success rate; interquartile range <30% of the median). Spleen marking was performed with an ultrasound probe included within the device before SSM. 

### 2.4. Esophago-Gastroduodenoscopies (EGD)

EGD examinations were always performed by four experienced endoscopists (>20,000 procedures) with Olympus^®^ (Tokyo, Japan) gastroscopes (GIF-Q165, GIF-Q180). All exams were performed under general anesthesia with intravenous propofol in the presence of an anesthesiologist and two nurses. 

During EGD, the esophagus, stomach and duodenum were carefully observed using conventional white light, and high definition techniques were used in doubtful cases (narrow-band imaging or magnification endoscopy). 

Esophago-gastroduodenal lesions were classified into erythema, erosions, ulcers, atrophy, metaplasia and polyps. Esophageal varices were classified according to the Baveno VII guidelines [11]: low-risk varices (thickness < 5 mm) and high risk varices (thickness > 5 mm or varices of any size and red wales or cherry red spots). The presence of portal hypertensive gastropathy was evaluated according to McCormack classification [34].

During each EGD, according to the updated Sydney protocol, two biopies within 3 to 5 cm from the pylorus (one in the lesser curvature and one in the greater curvature), one in the lesser curvature of the incisura angularis, and two biopies in the proximal corpus (one in the lesser curvature and one in the greater curvature) were taken [35]. Additional biopsies were taken in cases of a gastric lesion. If pre-malignant lesions were detected, all patients were counselled to follow up according the MAPS II guidelines [36].

### 2.5. Helicobacter Pylori Detection and Pre-Malignant Gastric Lesions Diagnosis

All biopsies were observed and classified by the same two pathologists.

Chronic gastritis was characterized according to the Sydney System grading for chronic gastritis [37]. Chronic inflammation was classified as mild, moderate or severe; activity was classified as mild (less than one third of pits and the surface infiltrated by neutrophils), moderate (one third to two thirds) or severe (more than two thirds); atrophy was classified into mild, moderate or severe and intestinal metaplasia as mild (less than one third of the mucosa involved), moderate (one third to two thirds) and severe (more than two thirds).

In addition to hematoxylin and eosin, modified Giemsa staining was used in all biopsy samples for *H. pylori* identification [38].

### 2.6. Statistical Analysis

The data were analyzed using SPSS 27.0 (IBM Corp, Armonk, NY, USA). 

The variables’ normality was evaluated using histograms and a Shapiro–Wilk test. 

Continuous variables are expressed as median (range) and categorical variables are reported as absolute (n) or relative frequencies (%). Analysis of variance was used to compare the differences in variable between groups. Group comparisons of categorical variables were analyzed with an X^2^ test or Fisher’s exact test. Group comparisons of continuous variables were analyzed with Student’s t-test for variables with a normal distribution and a Mann–Whitney U test for variables with a non-normal distribution.

Univariable analysis was performed using an independent samples t-test or independent samples Mann–Whitney U test for continuous variables and a Qui-scare test for categorical variables. For SSM, we performed a receiver operating characteristic (ROC) curve and calculated the area under the curve (AUROC). The ideal cut-off value for each ROC curve analysis was calculated using the Kolmogorov–Smirnov test. Sensitivity, specificity, positive and negative predictive values, positive and negative likelihood ratios, and diagnostic accuracy were calculated based on the ROC curve.

*p* values < 0.05 were considered significant. 

### 2.7. Ethical Considerations

This study was conducted in accordance with the Declaration of Helsinki and informed consent was obtained from all participants for both elastography and EGD. The study was approved by the Ethics Committee of Centro Hospitalar São João.

## 3. Results

### 3.1. Study Population

During the two-year study period (December 2020–December 2022), 155 patients were enrolled. 

The mean age was 64.1 years (±8.8) and 81.3% were male. The diagnosis of cirrhosis was based on LSM in the vast majority of the patients (73.5%) and the most common etiologies were alcoholic liver disease (72.9%), NAFLD (7.7%) and HCV (7.7%). All HCV patients were in sustained virologic response at enrollment. Most of the patients were classified as Child A (81.9%), 14.2% were classified as Child B and 3.9% were classified as Child C. Forty-five patients (29.0%) had previous episodes of cirrhosis decompensation. There were no patients with a family history of gastric cancer. There were 10 patients (6.5%) with hepatocellular carcinoma.

Clinical characteristics are displayed in Table 1.

### 3.2. LSM and SSM

The median value of LSM was 24.4 kPa [3.1–75.0] and of SSM was 49.1 kPa [12.8–100.0]. SSM was not possible due to anatomical reasons in five patients (3.2%)

LSM and SSM are depicted in Table 2. The median time between EGD and LSM and SSM was 3.0 months [0–5.0].

### 3.3. H. pylori Infection

*H. pylori* infection was diagnosed in 54 patients (34.8%). In the vast majority of the cases, *H. pylori* was detected in both the antrum and corpus simultaneously (75.9%), only in the antrum in seven cases (13.0%) and only in the corpus in six (11.1%).

**Figure 2:**
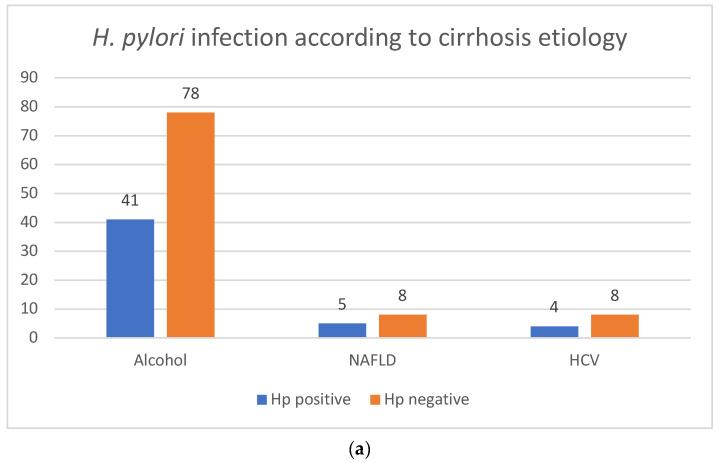

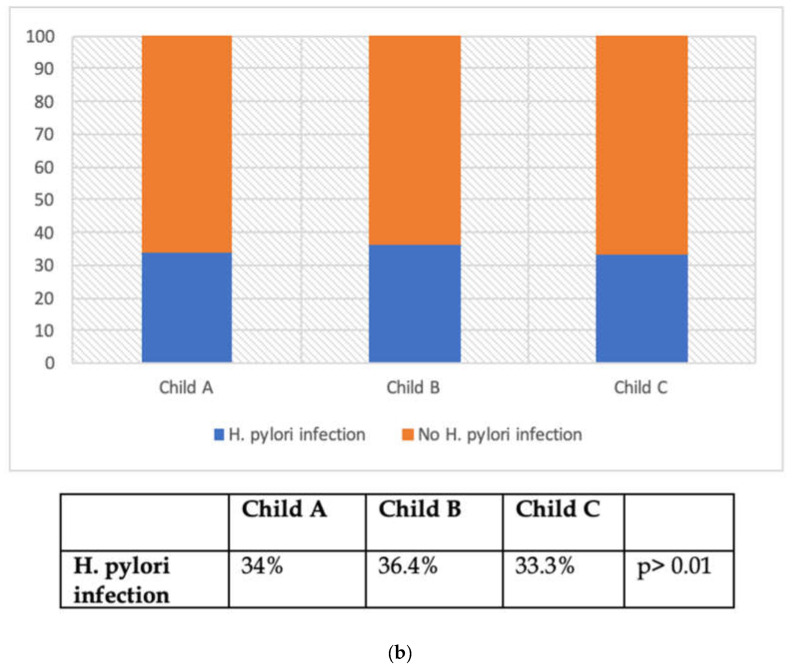
(**a**) *H. pylori* infection according to cirrhosis etiology. Legend: HCV: hepatitis C virus; NAFLD: non-alcoholic fatty liver disease. (**b**) *H. pylori* infection according to Child–Pugh classification.

**Figure 3:**
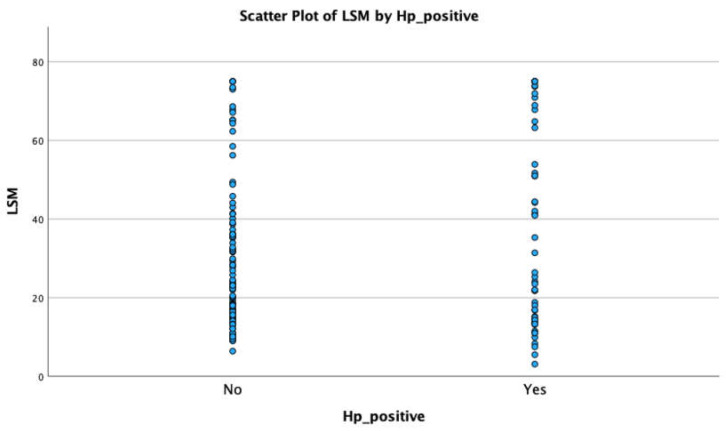

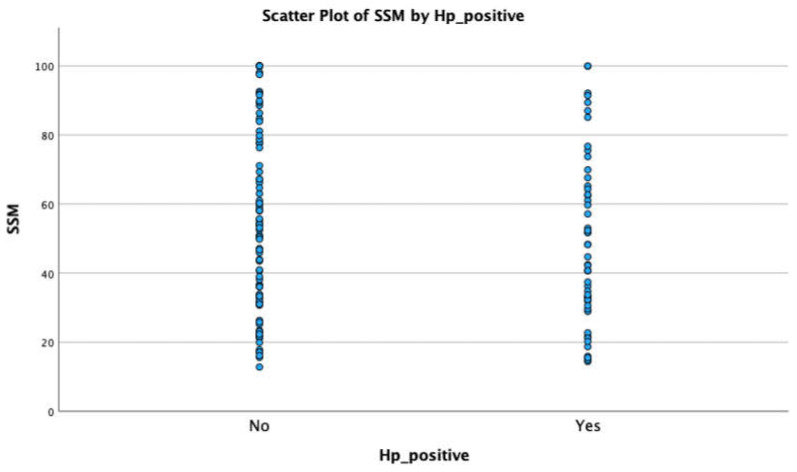
Relationship between LSM and SSM and *H. pylori* infection.

There were no statistically significant differences between the prevalence of *H. pylori* infection prevalence, the etiology of liver cirrhosis (Figure 2a) and the Child–Pugh classification (Figure 2b). There were also no statistically significant differences between the prevalence of *H. pylori* infection prevalence and LSM and SSM, and there were also no differences between the mean values of LSM and SSM in *H. pylori*-positive and -negative groups (Figure 3).

The presence of gastro-esophageal varices was comparable in *H. pylori*-positive and-negative groups, but we found a relationship between *H. pylori* infection and the presence of portal hypertension gastropathy (PHG) (*p* = 0.021). 

### 3.4. Endoscopic and Histologic Findings

Concerning the endoscopic findings, 50.3% presented esophageal varices (23.2% low-risk varices and 27.1% high-risk varices) and 32.9% presented portal hypertensive gastropathy. 

We found atrophy in 4.5% and metaplasia in 1.9% in the fundus and body, and atrophy in 1.9% and metaplasia in 7.1% in the antrum and incisura (Table 3a). We did not find any cases of dysplasia or gastric cancer.

Regarding histologic findings, fifty-five patients (35.5%) had gastric atrophy (OLGA 1—58.2%, OLGA 2—32.7%, OLGA 3—7.2% and OLGA 4—1.8%) and 38.7% had gastric metaplasia (OLGIM 1—63.3%, OLGIM 2—26.7%, OLGIM 3—6.7% and OLGIM 4—3.3%) (Table 3b). 

We did not find any relationship between endoscopic and histologic findings and the Child–Pugh classification or cirrhosis etiology.

There were no statistical differences in the prevalence of atrophy or metaplasia and the value of LSM and SSM. However, we found a relationship between SSM values and the presence of portal gastropathy (*p* < 0.001) and gastro-esophageal varices (*p* < 0.001).

In the subgroup analysis of patients with PHG, we found a higher prevalence of gastric atrophy (35.3%) and metaplasia (31.4%) when compared to patients without PHG, although this was not statistically significant.

## 4. Discussion

The association between *H. pylori* infection and presence and the severity of liver cirrhosis in cirrhotic patients has been debated over the years. A meta-analysis that included 21 studies worldwide showed a higher prevalence of *H. pylori* in cirrhotic patients, particularly in Europe and America [39]. However, when subgroup analysis was performed based on the etiology of liver cirrhosis, a higher prevalence was found in viral hepatitis and PBC, while in alcoholic cirrhosis, the prevalence of *H. pylori* infection was not statistically different from controls [39]. Several authors have reported a disappointing performance of serologic tests for *H. pylori* in cirrhosis, with a sensitivity of 89% and a specificity of 56%. This could be due to a possible interaction between the hypergammaglobulinemia of cirrhosis and the antibodies detected in the commercial kits [19,26,40]. In our study, we found a slightly lower prevalence of *H. pylori* infection (34.8%), which could be explained by two main reasons: (1) the principal etiology of cirrhosis was alcohol, which has previously been found not to be linked to a higher prevalence of *H. pylori;* (2) histology was used as the only method to evaluate *H. pylori* infection, as it is associated with higher sensitivity and specificity and is not affected by previous eradication, while the majority of other studies considered anti-*H. pylori* IgG-ELISA. Serology is noninvasive, inexpensive and easy to perform, making it useful for large-scale population studies, but it presents a high rate of false-positive results [41].

In view of the association between *H. pylori* infection and the severity of liver cirrhosis, we found a similar prevalence irrespective of the Child–Pugh score (*p* > 0.05), which is consistent with findings from several other authors [27,42,43].

There have been conflicting results from studies evaluating the relationship between *H. pylori* infection and NAFLD, with some reporting higher controlled attenuated parameters in *H. pylori*-infected patients and a reduction after successful eradication [44,45,46]. However, we did not find any differences in *H. pylori* infection according to the fibrosis stage evaluated by LSM [46].

To our knowledge, our study is the first to evaluate the association between LSM and SSM and *H. pylori* infection. Nevertheless, we were unable to find a significant association between *H. pylori* infection and surrogate markers of liver fibrosis and portal hypertension. In agreement with this finding, we also did not find a relationship between *H. pylori* infection and the presence of gastroesophageal varices, suggesting that they could represent two independent entities in cirrhosis [19,22]. On the other hand, the impact of *H. pylori* on PHG is still questionable. A meta-analysis including 13 studies did not find a relationship between *H. pylori* and the presence and severity of PHG, while other studies found a significant association, with *H. pylori* being more common in patients with severe PHG than in milder PHG [22,47,48]. In our study, we found a relationship between *H. pylori* infection and the presence of portal hypertension gastropathy (*p* = 0.021). This could be attributed to the changes in the stomach mucosa in cirrhosis and PHG: (1) a reduced mucus layer, (2) gastric mucosa swelling and hemorrhagic congestion, causing high inducible nitric oxide, and (3) a higher pH due to decreased acid secretion and prostaglandins. These factors weaken the gastric mucosal barrier, providing a suitable environment for *H. pylori* infection [47,49].

As expected, we found a significant relationship between SSM values and the presence of portal gastropathy (*p* < 0.001) and gastro-esophageal varices (*p* < 0.001), as they are a consequence of the development of portal hypertension.

Regarding the histologic findings, we observed atrophy in 4.5% and metaplasia in 1.9% in the fundus and body, and atrophy in 1.9% and metaplasia in 7.1% in the antrum and incisura. Although cirrhotic patients are recognized to have a higher risk of gastric cancer, we did not find a different prevalence of pre-malignant gastric lesions from the general population, as studies from developed countries report a prevalence ranging from 2% to 10.8% [50,51,52,53,54]. These findings suggest that, in cirrhotic patients, the higher risk of gastric cancer might be due to an accelerated carcinogenesis pathway rather than a higher prevalence of pre-malignant gastric lesions. Carcinogenesis in cirrhotic patients may be associated with congestive gastropathy, which can significantly facilitate the proliferation of epithelial cells in gastric mucosa, and also with zinc deficiency, which plays a central role in the growth, differentiation and metabolism of cells [53,54,55,56].

There were no statistical differences in the prevalence of atrophy or metaplasia and the value of LSM and SSM, as well as in relation to the Child–Pugh classification or cirrhosis etiology. This suggests that the grade of portal hypertension, the severity of liver disease and the etiology do not increase the risk of developing pre-malignant gastric lesions. 

To the best of our knowledge, and as one of the major strengths of our work, this is the first study to evaluate the prevalence of pre-malignant gastric lesions in cirrhotic patients and its relationship with portal hypertension evaluated through non-invasive tools. Additionally, we assessed the relationship between *H. pylori* infection and portal hypertension. Another positive aspect, compared to previous studies, is that *H. pylori* infection was diagnosed via histology, which has higher sensitivity and specificity. 

This study has significant limitations, including being a single-center study and including a small number of patients. Additionally, we lack information regarding *H. pylori* eradication after the diagnosis.

## 5. Conclusions

In conclusion, the prevalence of *H. pylori* infection and pre-malignant gastric lesions were not increased in cirrhotic patients and were not associated with LSM and SSM. On the other hand, we found an association between LSM and SSM and portal hypertensive gastropathy.

## Figures and Tables

**Table 1:** Characteristics of the 155 cirrhotic patients.

	*n* = 155
**Mean age (SD)**	64.1 years (±8.8)
**Male sex**	126 (81.3%)
**Cirrhosis diagnosis:** LSM Histological	114 (73.5%) 41 (26.5%)
**Cirrhosis etiology:** Alcohol NAFLD HCV Alcohol + HCV HBV Hemochromatosis Auto-immune hepatitis Drug-induced liver injury Primary biliary cholangitis PBC Alcohol + HBV	113 (72.9%) 13 (8.5%) 12 (7.7%) 6 (3.9%) 2 (1.3%) 2 (1.3%) 2 (1.3%) 2 (1.3%) 1 (0.6%) 1 (0.6%) 1 (0.6%)

Legend: HBV: hepatitis B virus; HCV: hepatitis C virus; LSM: liver stiffness measurement; NAFDL: non-alcoholic fatty liver disease; PBC: primary biliary cholangitis; SD: standard deviation.

**Table 2:** Median values of LSM, CAP and SSM of our population.

	*n* = 155
LSM (kPa)	24.4 [3.1–75.0]
IQR (%)	16% [0.0–30.0]
CAP (dB/m)	253.0 [100.0–394.0]
IQR (dB/m)	IQR 28.0 [0–145.0]
SSM (kPa)	49.1 [12.8–100]
IQR (kPa)	6.3 [0–39.0]

Legend: CAP: controlled attenuation parameter; IQR: inter-quartile range; LSM: liver stiffness measurement; SSM: spleen stiffness measurement.

**Table 3:** (**a**) Endoscopic findings. (**b**) Histological findings.

(a)	
**Stomach** **Fundus and body** Erythema Erosions Atrophy Metaplasia Portal hypertension gastropathy (mild/severe) ** Antrum and incisura** Erythema Erosions Ulcers Atrophy Metaplasia GAVE	20 (12.9%) 4 (2.6%) 7 (4.5%) 3 (1.9%) 51–45/6 (32.9%–29.0/3.9%) 106 68.4%) 29 (18.7%) 14 (9.0%) 3 (1.9%) 11 (7.1%) 2 (1.3%)
**(b)**	
**Stomach** ** Atrophy** Corpus and fundus Antrum and incisura OLGA 1 OLGA 2 OLGA 3 OLGA 4 ** Metaplasia** Corpus and fundus Antrum and incisura OLGIM 1 OLGIM 2 OLGIM 3 OLGIM 4	23 (14.8%) 41 (26.5%) 32 (58.2%) 18 (32.7%) 4 (7.2%) 1 (1.8%) 24 (15.6%) 52 (33.5%) 38 (63.3%) 16 (26.7%) 4 (6.7%) 2 (3.3%)

## Data Availability

No new data were created or analyzed in this study. Data sharing is not applicable to this article.

## Abbreviations

AFP	Alpha-fetoprotein
AIH	Autoimmune hepatitis
ALP	Alkaline phosphatase
ALT	Alanine aminotransferase
AST	Aspartate aminotransferase
CSPH	Clinically significant portal hypertension
EGD	Esophago-gastroduodenoscopy
GGT	Gamma-glutamyl transferase
HBV	Hepatitis B virus
HCV	Hepatitis C virus
*H. pylori*	*Helibobacter pylori*
HVPG	Hepatic venous pressure gradient
IL 1B	Interleucin 1B
IL 8	Interleucin 8
IL 10	Interleucin 10
INR	International normalized ratio
IQR	Interquartile range
LSM	Liver stiffness measurement
MALT	Mucosa-associated lymphoid tissue
MRI	Magnetic resonance imaging
NAFLD	Non-alcoholic liver disease
OLGA	Operative Link on Gastritis Assessment
OLGIM	operative link on intestinal metaplasia assessment
PHG	Portal hypertension gastropathy
PLT	Platelet
PBC	Primary biliary cholangitis
SSM	Spleen stiffness measurement
VEGF	Vascular endothelial growth factor
